# Calcium Intake Pattern among Japanese Women across Five Stages of Health Behavior Change

**DOI:** 10.2188/jea.17.45

**Published:** 2007-04-10

**Authors:** Yuan Zhang, Toshiyuki Ojima, Chiyoe Murata

**Affiliations:** 1 Department of Community Health and Preventive Medicine, Hamamatsu University School of Medicine.

**Keywords:** Transtheoretical Model, Calcium, Dietary, Osteoporosis, Japan, traditional Japanese diets

## Abstract

**BACKGROUND:**

The transtheoretical model (TTM) of health behavior change is one of the most promising approaches for health professionals to help individuals change their behaviors. Few studies have assessed calcium intake using the model on Asian women. This study aims at clarifying characteristics of each behavioral stage among Japanese women and providing clues to increase calcium intake to prevent osteoporosis.

**METHODS:**

A cross-sectional survey was conducted from September through November, 2005 using self-administered questionnaires. A total of 226 participants in an osteoporosis screening program were invited to take part in the study, and 150 women were enrolled.

**RESULTS:**

Adjusted means of total dietary calcium were positively significantly associated with successive stages (p<0.001). The proportion of calcium intake from plants and fish was higher in the precontemplation, contemplation, and preparation stages compared with the action and maintenance stages (p=0.038). Concomitantly, the plants and fish food group contributed 46.7% of total dietary calcium, while 32.4% was derived from milk and dairy food, and 20.9% from other foods. The correlation coefficient (95% confidence interval) between the proportion of calcium obtained from plants and fish and the proportion of fat energy was -0.22 (-0.37, -0.06).

**CONCLUSIONS:**

The proportion of calcium intake from plants and fish was higher among women in the lower stages compared with higher stages. Given the higher prevalence of lactose intolerance, it would seem plausible to recommend lower-stage women be educated and encouraged to derive more calcium from plants and fish diets as a means to prevent osteoporosis.

Osteoporosis and osteoporotic fractures have become a major health problem and an enormous economic burden in Japan and Asian countries as well as in other developed countries.^1^ With the lengthening of the life span, the number of patients with osteoporosis has been estimated to be over 10 million people now in Japan.^2^ The number of patients with femoral neck fracture has nearly doubled over the 15 years from 1987 through 2002.^3^ Because the elderly population in Japan is rapidly increasing, prevention of osteoporosis presents an urgent issue.^4^

Inadequate dietary calcium intake has been reported to be one of the risk factors for accelerating bone loss and may contribute to osteoporosis in later years.^5^^-^^9^ According to results from the 2003 National Health and Nutrition Survey in Japan, women aged 30 or over were not meeting the dietary goal of calcium intake.^10^ That dietary goal for women 18-69 years old was set at 600 mg/day by the Ministry of Health, Labour and Welfare.^11^

Compared to the United States and Europe, the plausible reason for the low intake of calcium in Asia and Africa is low consumption of milk and dairy products.^12^ Milk is not as popular in Japan as in Western societies, one of the reasons for which may be the high prevalence of lactose intolerance among Japanese.^13^^,^^14^ Japanese are accustomed to traditional Japanese dishes such as soy products and fish. Although some studies have reported that such a diet has many important antiatherosclerotic^15^ and antithrombotic effects,^16^ the intake of soy and fish is reportedly beneficial in improving bone density among adolescents.^17^ Tsuchida et al^18^ revealed that soybean intake has a significantly positive association with bone mineral density in middle-aged Japanese women, suggesting that bone mineral density might be more beneficially affected by a variety of dietary calcium sources rather than relying on the total amount of calcium intake alone. The traditional Japanese diet is still thought to be one of the important dietary patterns for Japanese women.

This study has been guided by the transtheoretical model of health behavior change, which is a psychosocial model of behavior change developed by Prochaska and colleagues.^19^ The trans-theoretical model is an innovative and comprehensive framework that conceptualizes when and how behavior change occurs. The stages of change construct, the core of the transtheoretical model, proposes that behavior change is a dynamic process with five distinct stages. The transtheoretical model appears to be one of the more promising recent approaches used by health professionals trying to assist individuals change their dietary behavior. One appealing aspect of the model is that interventions can be tailored to the individual's readiness to change behavior. In Western countries, several studies that investigated the stages of dietary behavior change have focused on the goals of increasing calcium intake,^20^^,^^21^ lowering fat intake,^22^ and increasing fruit and vegetable consumption.^23^ Few studies, however, have assessed calcium intake behavior using transtheoretical model on Asian women.

Therefore, we conducted an investigation to clarify characteristics of each behavioral stage among Japanese women and provide clues that might help them to increase calcium intake to prevent osteoporosis.

## METHODS

### Study Design and Subjects

A cross-sectional study was conducted in Fukuroi City, Shizuoka Prefecture, Japan from September through November 2005. An osteoporosis screening program established by the local government has been provided at the city hospital. The program consisted of osteoporosis screening and health education. The bone mineral density results were given back to the participants at the time of health education which took place 2-4 weeks after the screening, depending on the individual participant. The program participants have been recruited by governmental newsletter or posters. Though residents of the city aged 30 or over who wished to participate in the program have been accepted, women with a specific age having a last digit of 0, 3, 5, or 7 between 40-77 years old had been encouraged by the health center to participate.

A total of 226 program participants were invited to participate in the present study before the screening test. All of them could walk unaided and were apparently healthy. Among them, 167 (73.9%) volunteers consisting of 4 men and 163 women consented to participate in the study. Of these, 13 women failed to respond to all the items on food frequency in the questionnaire. The men were excluded from the analyses because of the small number to simplify the interpretation of the results. Finally, 150 women were enrolled in the study.

This study has been approved by the Fukuroi City Health Center, the administration committee of Fukuroi City Hospital, and the institutional review board of the Hamamatsu University School of Medicine.

### Data Collection

Each participant was requested to fill out a self-administered questionnaire. The set of questionnaires for this study consisted of basic questions, the osteoporosis knowledge test, the osteoporosis self-efficacy scale, the stage of change for calcium intake, the food frequency questionnaire based on food groups, and the osteoporosis health belief scale.

Basic questions included age, age at menarche, menopausal status, age at menopause, number of parity, bone mineral density screening experience, acquisition of information regarding osteoporosis prevention from health centers, and regular physical activity.

We used a single question to assess participants' current stage of change in calcium intake behavior. Questions were derived from the report by Marsden.^24^ Stages were determined by asking "Which of the following statements best describes your thoughts on calcium intake?" (1) "I have never thought about increasing the amount of calcium I consume, and I do not intend to do so within the next 6 months" (precontemplation); (2) "I have thought about increasing the amount of calcium I consume sometime within the next 6 months" (contemplation); (3) "I am thinking about increasing the amount of calcium I consume in the next month" (preparation); (4) "I have been increasing the amount of calcium intake for less than 6 months" (action); (5) "I have been increasing the amount of calcium intake for more than 6 months" (maintenance).

The food frequency questionnaire based on food groups^25^ was used to estimate the amount of calcium intake of various food groups and other nutrient intake. It featured questions about individual intake of 29 foods and 10 types of cooking during the past 1-2 months. Takahashi et al^25^ have validated the food frequency questionnaire based on food groups and showed it was a useful instrument. Food groups were re-categorized into 3 groups for the study. The first group was "plants and fish," which consists of sources of calcium intake other than milk and dairy food. The second group was "milk and dairy food," which had usually been the most recommended to increase calcium intake. The third group was "other foods," which had not been recommended to increase calcium intake.

The osteoporosis knowledge test, the osteoporosis self-efficacy scale, and the osteoporosis health belief scale were developed by Kim et al.^26^^,^^27^ We eliminated one osteoporosis knowledge test and two osteoporosis health belief scale questions because they were not appropriate for Japanese people.

The osteoporosis knowledge test, which we finally used, contained 23 multiple-choice questions on osteoporosis prevention knowledge. Questions related to dietary calcium and exercise domains. Scores had a possible range of 0 to 16 for calcium domain and 0 to 15 for exercise domain. Higher scores indicated a better knowledge level.

The osteoporosis self-efficacy scale was a 12-item scale for self-efficacy. Subjects were asked to rate 5-point scale questions. The tool consisted of two subscales, calcium intake and exercise. Scores had a possible range of 0 to 24 for each subscale. Higher scores indicated participants' confidence in pursuing behaviors that would help prevent osteoporosis.

The osteoporosis health belief scale used was a 40-item tool consisting of 7 subscales for health belief. Each item was rated by the subject using a 5-point scale. Scores had a possible range of 0 to 24 for each subscale except a possible range of 0 to 16 for susceptibility. A higher score indicated a higher tendency for each subscale item.

In addition to self-administered questionnaires, the bone mineral density of the lumbar spine L2-L4 was measured by a dual-energy X-ray absorptiometer (DXA) (QDR-2000; Hologic). All subjects were measured with the same densitometer operated by the same trained technician. The coefficient of variation (CV) value for the lumbar spine bone mineral density measurements was 1.0%. The bone mineral density was calculated as bone mineral content (g) / bone area (cm^2^).

Height and weight were measured at the time of the bone mineral density measurements. Height was measured to the nearest 0.5 cm, and weight to the nearest 0.1 kg. Body mass index (BMI) was calculated as the ratio of weight in kilogram to squared height in meters.

### Statistical Analysis

Descriptive statistics were used to summarize participants' demographic variables, osteoporosis-related knowledge, health belief and self-efficacy. Trend tests have been conducted using simple linear regression models for these variables in Table 1.

**Table 1.** Demographic variables and osteoporosis-related knowledge, self-efficacy, and health belief scales across 5 stages.

Variables	Total	Precontemplation	Contemplation	Preparation	Action	Maintenance	Trend test p-value**^§^**
Total participants^*^	150 (100%)	10 (7%)	12 (8%)	65 (43%)	36 (24%)	27 (18%)	

Demographic variables							
Age^†^	58.8 (9.4)	55.1 (12.5)	56.3 (11.0)	59.1 (9.5)	59.5 (8.1)	59.4 (9.1)	0.228
Age at menarche^†^	13.7 (1.5)	15.0 (2.2)	14.4 (1.1)	13.5 (1.3)	13.5 (1.5)	13.4 (1.3)	0.012
Years after menopause^†^	11.7 (6.7)	14.7 (8.4)	13.4 (6.1)	11.9 (7.1)	10.9 (6.2)	10.9 (6.4)	0.132
Body Mass Index (BMI) (kg/m^2^)^†^	21.8 (3.0)	22.4 (3.9)	21.2 (1.4)	21.9 (3.3)	21.1 (2.7)	22.4 (2.7)	0.992
Bone Mineral Density (BMD) (g/cm^2^)^†^	0.86 (0.16)	0.73 (0.17)	0.83 (0.11)	0.83 (0.13)	0.89 (0.19)	0.96 (0.16)	<0.001
Parity^†^	2.4 (1.2)	1.6 (0.5)	1.8 (0.4)	2.3 (1.3)	2.6 (1.2)	3.1 (1.3)	<0.001
Prevalence of osteoporosis or osteopenia^‡^	49 (33%)	5 (50%)	3 (25%)	26 (40%)	12 (33%)	3 (11%)	0.029
Proportion of menopause^‡^	116 (77%)	6 (60%)	7 (58%)	53 (82%)	28 (78%)	22 (82%)	0.196
Regular physical activity^‡^	43 (28.7%)	0 (0%)	3 (25.0%)	13 (20.0%)	12 (33.3%)	15 (55.6%)	<0.001

Obtaining information regarding osteoporosis from health centers^‡^	51 (34%)	1 (10%)	1 (8%)	16 (25%)	17 (47%)	16 (59%)	<0.001

Osteoporosis Knowledge Test (OKT)							
Osteoporosis Knowledge Test Calcium^†^	11.4 (3.3)	10.3 (3.2)	9.4 (3.3)	11.2 (3.1)	12.0 (3.1)	12.4 (3.1)	0.003
Osteoporosis Knowledge Test Exercise^†^	8.9 (2.7)	7.9 (2.1)	7.5 (3.0)	9.0 (3.0)	9.2 (2.6)	9.4 (2.2)	0.038

Osteoporosis Self-Efficacy Scale (OSES)							
Osteoporosis Self-Efficacy Scale Calcium^†^	11.7 (3.6)	10.2 (3.6)	9.8 (4.0)	11.4 (3.5)	12.6 (3.4)	13.1 (3.4)	<0.001
Osteoporosis Self-Efficacy Scale Exercise^†^	11.8 (4.0)	10.6 (3.8)	10.1 (4.2)	11.5 (3.9)	12.4 (4.2)	13.0 (3.9)	0.012

Osteoporosis Health Belief Scale (OHBS)							
Susceptability^†^	11.2 (3.2)	9.4 (2.3)	9.2 (3.9)	11.0 (3.1)	11.7 (2.8)	12.7 (3.0)	<0.001
Seriousness^†^	16.0 (3.4)	15.5 (2.3)	14.3 (3.0)	16.2 (3.5)	16.4 (3.7)	16.2 (3.5)	0.231
Benefits-Calcium^†^	11.6 (3.8)	10.6 (3.8)	10.1 (4.2)	11.5 (4.0)	11.9 (3.5)	12.5 (3.3)	0.050
Benefits-Exercise^†^	18.3 (2.9)	17.6 (2.9)	17.1 (2.3)	18.0 (3.2)	18.7 (2.4)	19.3 (2.5)	0.007
Barriers-Calcium^†^	8.2 (2.5)	9.1 (4.3)	9.5 (2.5)	8.3 (2.2)	7.8 (2.4)	7.4 (2.1)	0.006
Barriers-Exercise^†^	8.3 (2.7)	9.1 (4.3)	9.5 (2.5)	8.7 (2.7)	7.8 (2.4)	7.4 (2.1)	0.004
Health Motivation^†^	15.5 (3.2)	13.8 (4.2)	13.8 (3.0)	15.4 (3.4)	15.7 (2.3)	16.9 (2.8)	0.001

Participants with calcium intake < 600 mg/day^‡^	95 (63%)	9 (90%)	10 (83%)	57 (88%)	16 (44%)	3 (11%)	<0.001

Daily calcium supplement intake^‡^	21 (14%)	1 (10%)	3 (25%)	4 (6%)	7 (19%)	6 (22%)	0.153

Total energy intake (kcal)^†^	1665 (395)	1372 (364)	1437 (318)	1557 (289)	1723 (304)	2057 (475)	<0.001

* : N (proportion to total participants)

† : Mean (standard deviation)

‡ : N (proportion to participants in respective stage)

**§** : Trend tests have been conducted using simple linear regression

We calculated dietary calcium intake from various food groups across 5 stages in order to clarify calcium intake pattern. We used multiple linear regression models for trend testing^28^ and for calculating means of calcium intake adjusting for age and total energy across 5 stages. We transformed the categorical variable consisting of 5 stages into numerical values by calculating mean ranks of women located in each stage, which were 5.5, 16.5, 55.0, 105.5, and 137.0, respectively. We entered them into the models as dependent variables.

Correlation coefficients (r) and their 95% confidence intervals (CI) were used to determine the relationships between the proportion of calcium obtained from plants and fish (or milk and dairy food) and the proportion of fat energy (or age) in scatter diagrams.

Statistical analysis was carried out using SPSS^®^ for Windows version 12.0.

## RESULTS

The 150 participants were grouped into 5 stages of change. Table 1 shows demographic variables and osteoporosis-related knowledge, self-efficacy, and health belief scales. The mean of bone mineral density was 0.86 g/cm^2^, and the bone mineral density in the precontemplation stage was its lowest at a level of 0.73 g/cm^2^. Variables including age at menarche, bone mineral density, number of parity, and regular activity level were positively and significantly associated with successive stages. A similar association existed about obtaining information regarding osteoporosis from health centers, osteoporosis-related knowledge, self-efficacy, susceptibility, benefit, and health motivation. Barriers to calcium intake and exercise were negatively and significantly associated with successive stages. The proportions of participants with calcium intake less than 600 mg/day among participants in the respective 5 stages were negatively and significantly associated with their stages (p<0.001).

Table 2 mainly shows adjusted means of dietary calcium obtained from various food groups across the 5 stages. The mean of total dietary calcium was 574.0 mg/day. A significant positive association was observed between total dietary calcium intake and the stages of change (p<0.001). In order to assess calcium intake, we simplified food groups into 3 categories. Plants and fish contributed 46.7% of total dietary calcium, while 32.4% was derived from milk and dairy food, and 20.9% from other foods. The proportion of calcium obtained from soy was 17.2% of total dietary calcium, the highest in the plants and fish group. The adjusted means of calcium intake for women in the precontemplation, contemplation, and preparation stages were below the dietary goal of 600 mg/day, against 635.7 mg/day in the action stage and 697.8 mg/day in the maintenance stage which met the dietary goal of 600 mg/day.^11^

**Table 2.** Adjusted dietary calcium obtained from various food groups across 5 stages.

Variables	Total	Precontemplation	Contemplation	Preparation	Action	Maintenance	Trend test p-value	

							Means	Proportion
Total dietary calcium (mg/day)*	574.0 (100)	435.5 (100)	457.6 (100)	533.6 (100)	635.7 (100)	697.8 (100)	<0.001	
(1) Plants and fish*	267.9 (46.7)	224.3 (51.5)	231.3 (50.5)	255.5 (47.9)	287.9 (45.3)	307.6 (44.1)	0.004	0.041
Small fish*	28.4 (4.9)	20.8 (4.8)	22.1 (4.8)	26.4 (4.9)	32.2 (5.1)	35.7 (5.1)	0.031	0.859
Fish*	19.9 (3.5)	20.3 (4.7)	20.3 (4.4)	20.3 (3.8)	20.1 (3.2)	20.2 (2.9)	0.927	0.002
Seaweed*	7.6 (1.3)	5.6 (1.3)	5.8 (1.3)	6.5 (1.2)	7.5 (1.2)	8.1 (1.2)	0.214	0.452
Cereal*	23.2 (4.0)	20.4 (4.7)	20.7 (4.5)	21.8 (4.1)	23.2 (3.6)	24.1 (3.5)	0.331	0.924
Soy*	98.5 (17.2)	71.7 (16.5)	76.1 (16.6)	91.2 (17.1)	111.4 (17.5)	123.7 (17.7)	0.002	0.007
Green and yellow vegetables*	33.3 (5.8)	29.8 (6.8)	30.2 (6.6)	31.7(5.9)	33.8 (5.3)	35.0 (5.0)	0.376	0.223
Light-colored vegetable*	29.3 (5.1)	29.4 (6.8)	29.5 (6.4)	29.8 (5.6)	30.2 (4.8)	30.5 (4.4)	0.871	0.080
Potato*	8.9 (1.6)	8.7 (2.0)	8.7(1.9)	8.9 (1.7)	9.3 (1.4)	9.3 (1.3)	0.741	0.041
Beans*	9.7 (1.7)	8.4 (1.9)	8.6 (1.9)	9.5 (1.8)	10.8 (1.7)	11.5 (1.6)	0.247	0.919
Fruits*	9.0 (1.6)	9.2 (2.1)	9.3 (2.0)	9.4 (1.7)	9.4 (1.5)	9.5 (1.4)	0.895	0.040

(2) Milk and dairy food*	186.2 (32.4)	68.4 (15.7)	87.1 (19.0)	151.8 (28.4)	238.6 (37.5)	291.4 (41.8)	<0.001	<0.001
Milk*	117.6 (20.4)	29.7 (6.8)	43.5 (9.5)	91.4 (17.1)	155.7 (24.5)	194.8 (27.9)	<0.001	<0.001
Dairy food*	68.6 (12.0)	38.7 (8.9)	43.6 (9.5)	60.4 (11.3)	82.9 (13.0)	96.6 (13.8)	<0.001	0.039

(3) Other foods*	119.8 (20.9)	142.8 (32.8)	139.2 (30.4)	126.3 (23.7)	109.2 (17.2)	98.8(14.1)	0.098	<0.001
Meat and Egg*	14.4 (2.5)	15.1 (3.5)	15.1 (3.3)	14.7 (2.8)	14.4 (2.3)	14.3 (2.0)	0.703	<0.001
Pickles*	27.4 (4.8)	28.2 (6.5)	28.2 (6.1)	28.1 (5.2)	27.8 (4.4)	27.6 (4.0)	0.898	0.009
Sweet and Sugar*	44.5 (7.8)	54.2 (12.4)	52.5 (11.5)	46.8 (8.8)	39.3 (6.2)	34.5 (4.9)	0.085	<0.001
Other*	33.5 (5.8)	45.3 (10.4)	43.4 (9.5)	36.7 (6.9)	27.7 (4.4)	22.4 (3.2)	0.385	0.143

* : Adjusted mean (proportion of adjusted mean of calcium intake from various food to total calcium intake in respective stage)

† : Trend tests and adjusted means have been calculated using multiple linear regression adjusting for age and energy

Dietary calcium does not include supplement

Parcentages in parentheses

The proportions of calcium from plants and fish were negatively and significantly associated with the stages (p=0.041). There was a significantly positive association between the proportion of calcium obtained from milk and dairy food and stages of change (p<0.001). Though the p-value is not shown in Table 2, the proportion of calcium from plants and fish in the group combining the precontemplation, contemplation, and preparation stages was significantly higher than that in the group combining the action and maintenance stages (p=0.038). On the other hand, the proportion of calcium obtained from milk and dairy food in the group of action and maintenance stages was significantly higher than that in the group of precontemplation, contemplation, and preparation stages (p<0.001).

**Figure 1.**
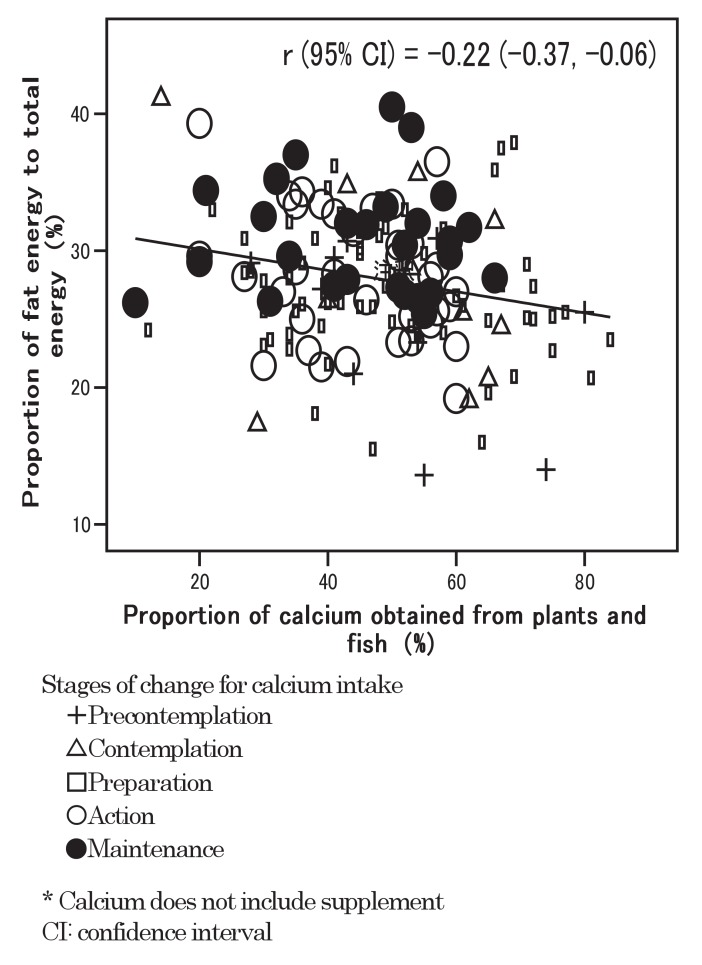
Relationship between proportion of calcium obtained from plants and fish and proportion of fat energy.

The relationship between the proportion of calcium obtained from plants and fish and the proportion of fat energy is shown in Figure 1. A negative association was observed and r (95% CI) was -0.22 (-0.37, -0.06). The figure shows that the participants in the maintenance stage distributed in the upper-left area, while those in the precontemplation stage distributed in the lower-right area.

**Figure 2.**
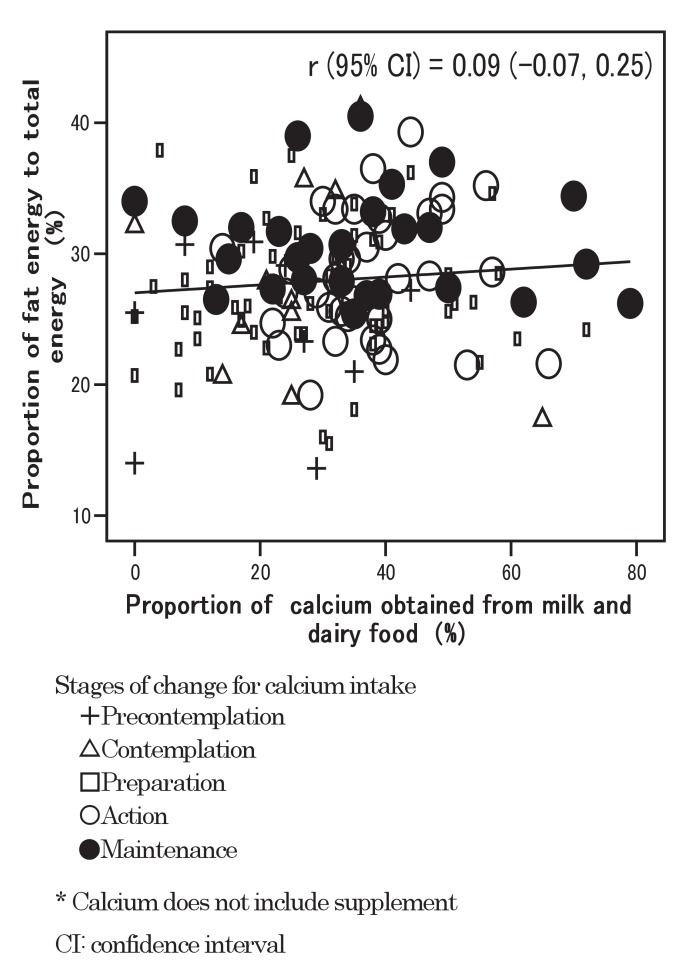
Relationship between proportion of calcium obtained from milk and dairy food and proportion of fat energy.

The relationship between the proportion of calcium obtained from milk / dairy food and the proportion of fat energy is shown in Figure 2; r (95% CI) was 0.09 (-0.07, 0.25); there was no clear association observed. Participants in the precontemplation stage distributed in the lower-left area, and those in the maintenance stage in the upper area.

**Figure 3.**
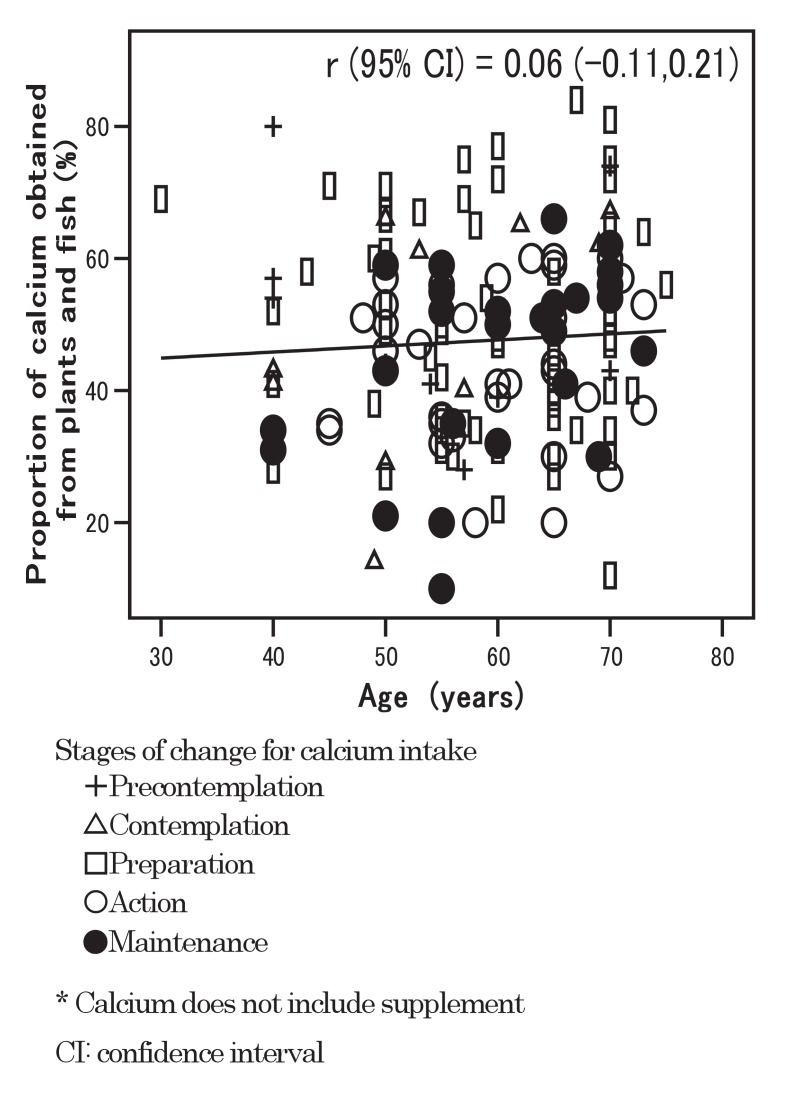
Relationship between age and proportion of calcium obtained from plants and fish.

The relationship between age and the proportion of calcium obtained from plants and fish is shown in Figure 3; r (95% CI) was 0.06 (-0.11, 0.21).

## DISCUSSION

In the current study, calcium intake rose with each progression to a higher stage. The adjusted means of calcium intake in the action and maintenance stages exceeded the dietary goal of 600 mg/day. Progressive advance of women from the precontemplation, contemplation, and preparation stages to the higher stages is important for them to have their calcium intake reach their dietary goal and prevent osteoporosis.

The proportion of calcium from plants and fish in the precontemplation, contemplation, and preparation stages was significantly higher than in the other two stages. The proportion of calcium from milk and dairy food was significantly higher in the action and maintenance stages in our study, possibly because the participants in those stages had increased calcium from milk and dairy food more than plants and fish compared to their counterparts in the 3 other stages. On the other hand, women in the precontemplation, contemplation, and preparation stages might have difficulties in consuming much milk and dairy food, although we can not be sure that those women had already tried to increase their milk and dairy food intake since such questions were not among the questionnaire items.

Although milk and dairy food have been thought highly effective in increasing calcium intake, some Japanese prefer the taste of traditional Japanese food to that of milk and dairy food.^17^^,^^29^ Moreover, the prevalence of lactose intolerance among Japanese and other Asians is much higher than among Caucasians.^13^^,^^30^ Though we did not ask this specifically in the current study, not a few people in the lower stages may have reservations about raising their milk and dairy food intake; they may find taking more calcium from plants and fish to be a more attractive option than milk and dairy food. Such recommendations may lower resistance to taking more calcium among people who do not like milk and dairy food.^29^

The recommendation to obtain more calcium from plants and fish may have some other advantages. The proportion of calcium obtained from plants and fish was negatively associated with the proportion of fat energy, while the proportion of calcium obtained from milk and dairy food was positively associated with it. Plants and fish foods are typical items in the traditional Japanese diet. There is much evidence to show that these traditional dietary habits in Japan may result in lower rates of coronary heart disease than in Western countries.^31^^-^^33^ Although the correlation was not so clear in the present results, Horwath et al^34^ have revealed that increasing milk product intake results in increased dietary fat and higher cholesterol levels. Consuming plant and fish foods might be preferable to milk and dairy food for decreasing fat intake. In our study, participants in the precontemplation stage distributed in the area with a low proportion of fat energy and a high proportion of calcium obtained from plants and fish. Such a distribution pattern was preferable to that in the maintenance stage, though calcium intake in the precontemplation stage was lower than in the maintenance stage. Egami et al^35^ disclosed associations of dietary sources of calcium including milk and dairy food, pulses, fish and shellfish, vegetables and fruits with bone mineral density in young Japanese adults. Providing health education programs to prevent osteoporosis, observing one's dietary intake pattern, and especially estimating one's calcium intake from plants / fish and milk /dairy food, may be useful.

We have been concerned that the recommended strategy of consuming more plants and fish may be attractive only for older people because Hirota et al^17^ have shown that traditional dietary habits in Japan have been gradually declining, especially in the younger generation. However, in Figure 3, proportions of calcium obtained from plants and fish are not so different between the younger and older generations. Even if some people prefer Western over traditional Japanese food, recommendations to consume more plants and fish would still be worth emphasizing to younger people.

In the current study, the total energy intake differed across the 5 stages (p<0.001). Therefore we have observed dietary calcium intake adjusting for energy and age. The proportion of women who were engaging in regular physical activity was significantly high among advancing stages. It may contribute to the significant difference in energy intake across the stages, and possible information bias by self-rating answers about food intake, even if the food frequency questionnaire based on food groups has been validated.^25^

Our data analysis also revealed that women in the action and maintenance stages have higher scores in terms of knowledge, self-efficacy for calcium intake, and health belief regarding osteoporosis compared with those in the precontemplation, contemplation, and preparation stages. These factors might be the determinants of placing participants in higher stages and resulting in increasing calcium intake. Although Cline and Worley^36^ did not take various stages into consideration as in their study, they found that people who believed themselves susceptible to osteoporosis and perceived the benefits to be obtained from taking calcium in fact took significantly more calcium.

This study has several limitations. First, the conclusions are speculative because it was a cross-sectional study and thus subject to alternative interpretations of causal order. Second, the sample size was limited. Third, the current study might contain selection bias because we did not use a random sampling method with all the residents. Fourth, information bias might occur in our study. One of the causes might be application of the self-administered food frequency questionnaire. Another might be possible changes in dietary habit with seasonal variations. In order to avoid such bias, the investigation might be conducted in another season using the dietary record method; however, we collected data only once to reduce the burden among participants. Furthermore, we did not elicit important information on matters such as the amount of calcium supplement taken and low-fat milk intake, which did not allow us to conduct further detailed analyses. Finally, there might be some confounding factors such as education levels or psychosocial factors not included in our questionnaires.

In conclusion, the present study revealed that the proportion of calcium from plants and fish was higher in the precontemplation, contemplation, and preparation stages compared with the action and maintenance stages. Given their lactose intolerance and distaste for milk and dairy products, not to mention the traditional Japanese diet, it would seem only natural to recommend that lower-stage women be educated and encouraged to derive more calcium from plants and fish diets as a means to prevent osteoporosis.
